# Hydrophobia

**Published:** 1869-04

**Authors:** 


### HALL'S
### JOURNAL OF HEALTH.

VOL. XVI. APRIL, 1869. No. 4.

### HYDROPHOBIA.

THE bite of a dog is as liable to be followed by what is called "hydrophobia" or fear of water, in winter as in summer.

The bite of a dog while being beaten or misused, inducing in him a feeling of irritation, anger, or excessive alarm, may give rise to hydrophobia in man, if the man is himself in a state of anger.

A few months ago a man commenced whipping a dog, and was bitten; his wife was bitten at the same time, but was a mere spectator. In a short time the man died, with the ordinary symptoms of hydrophobia, while his wife suffered no ill effect. An account of this was given in the June number of last year.

Within a week of this writing, Mr. Ludlam, a respectable and worthy citizen of Brooklyn, died of hydrophobia, who had been bitten four weeks before by a dog while he was whipping it; the wound healed rapidly. The dog had given no indications of madness before the whipping, nor afterwards.

### PHRENSY.

After the first few seconds have passed in battle, a kind of phrensy takes possession of the soldier, and he fears nothing.

Cases are given where a man has killed another, and yet after knowing that the victim was dead, continued hacking the unresisting body to pieces, or beating out the brains without being able to give any other reason for it than he seemed "possessed." Some parents may remember a feeling of increasing anger, of rising wrath, while chastising a child; and sometimes have so lost all sense of reason and judgment, as to continue the beating until the child was dead. A case of this kind occurred in this State not a great while ago, remembered by many who read this. Whether this phrensied condition of the mind has any influence on the physical condition of the blood or nervous fluid, rendering the body liable to certain symptoms, on certain conditions, has not as yet been determined; but one thing seems to be very certain, and every reader is interested in knowing the terrible fact that there is such a thing as

IMAGINARY HYDROPHOBIA, which is as terribly and as surely fatal as the more real form of the disease. Some years ago we gave a notice of a case of then recent occurrence, the facts being known to us personally, where a friend's gardener had been bitten a year before by a dog, but the wound had healed and had been forgotten, when the gardener was told that a friend of his was suffering from hydrophobia; within a few days the gardener died with all the generally believed symptoms of the disease.

It is an almost universal belief, that an inability to swallow water, or a horror of the attempt to do so, with the consciousness of an inability to do it, is so much a part of the disease, that when the symptom is observed the case is at once set down by everybody as hydrophobia, as a matter of course; but the actual fact is that dogs who suffer from real hydrophobia do not have a fear of water more than once in a hundred cases. Blaine observed it but once in several hundred cases, and Youatt only once or twice during his whole life.

When Mr. L. was taken ill, a month after the bite, and after the wound had entirely healed, he asked his physician if he thought he was in danger of "that terrible disease."

This shows that he had a dread of it; that his mind had been dwelling on the subject; that he was nervously sensitive on that point. Almost every one believes that a hydrophobic man shudders at the very sight of water, and that he will bite others if he can. We have seen that the fear of water is not a characteristic symptom of the disease; and we know, further, that men never attempt to bite others until they become deranged. Yet, Mr. L., believing that the fear of water was a symptom of the disease, could not swallow a drop; and, believing that the hydrophobic were inclined to bite others, he warned his family to keep away, lest he should bite them; thus he had the symptoms, in striking prominence, which he thought peculiar to the disease, but which were not necessarily any part of it; hence, it is clear that he died of a disease induced by the imagination. Only about one in twenty of those bitten by dogs actually mad become hydrophobic themselves. Children are attacked within thirty days after the bite; grown persons, who have greater power of resistance against the ill effects, are not attacked for several months.

Mr. John S. Roads, an aged and respectable citizen of Marblehead, died on Friday last from the effects of the bite of a cat, which happened four weeks before his death. He was attempting to punish the animal for scratching a child, when the animal bit him, and the bite resulted in his death as stated. Here a cat, under the irritation of a whipping, gives a fatal bite.

The fang of a rattlesnake is hollow; at the bottom of it is a sack or pouch which is filled with its venom, but this venom is ejected through the fangs into the wound which the fang first makes only when the snake is alarmed, irritated or angered.

After Mr. L. had whipped his dog, and had been bitten, he sat down on the sofa, and the animal, as if to make friends, came up to Mr. L. and crouched under the sofa behind his feet; a little child attempted to pull the dog out, and was bitten, but no ill result followed the bite.

These narrations seem to show that "hydrophobia," or rather death by convulsions, occurs in winter as well as in summer. That it follows the bites of dogs, cats and other animals. That a diseased imagination may cause death from convulsions. That animals not mad, may inflict wounds while in fear or anger, which will cause death by the same convulsions which end real hydrophobia. That dogs and cats are a nuisance any how. That there ought to be no dogs and cats. That if you want to wallop dogs and cats, it is better to do it at a distance with a long pole, and not be holding them up by the tail to be vexed and irritated. There are a good many people who would bite as vigorously as the animals, if treated similarly. But if you will have dogs and cats swarming around you, and will hold them up by their caudal extremities, and will whip them, and are bitten in return, why then "As ye mak your bed, so ye maun lie doon."

